# Recent Progress on the Research of 3D Printing in Aqueous Zinc-Ion Batteries

**DOI:** 10.3390/polym17152136

**Published:** 2025-08-04

**Authors:** Yating Liu, Haokai Ding, Honglin Chen, Haoxuan Gao, Jixin Yu, Funian Mo, Ning Wang

**Affiliations:** 1 Future Technology School, Shenzhen Technology University, Shenzhen 518118, China; 202201001108@stumail.sztu.edu.cn (Y.L.); hk_ding@outlook.com (H.D.); 202200202136@stumail.sztu.edu.cn (H.C.); 202200101080@stumail.sztu.edu.cn (H.G.); 202200404003@stumail.sztu.edu.cn (J.Y.); 2 Department of Materials Science and Engineering, Shenzhen Technology University, Shenzhen 518118, China

**Keywords:** zinc-ion batteries, 3D printing, electrodes, electrolytes, energy-density

## Abstract

The global transition towards a low-carbon energy system urgently demands efficient and safe energy storage solutions. Aqueous zinc-ion batteries (AZIBs) are considered a promising alternative to lithium-ion batteries due to their inherent safety and environmental friendliness. However, conventional manufacturing methods are costly and labor-intensive, hindering their large-scale production. Recent advances in 3D printing technology offer innovative pathways to address these challenges. By combining design flexibility with material optimization, 3D printing holds the potential to enhance battery performance and enable customized structures. This review systematically examines the application of 3D printing technology in fabricating key AZIB components, including electrodes, electrolytes, and integrated battery designs. We critically compare the advantages and disadvantages of different 3D printing techniques for these components, discuss the potential and mechanisms by which 3D-printed structures enhance ion transport and electrochemical stability, highlight critical existing scientific questions and research gaps, and explore potential strategies for optimizing the manufacturing process.

## 1. Introduction

With the global energy structure transitioning towards low-carbonization, efficient, safe, and low-cost energy storage technologies have become the key support for the large-scale application of renewable energy [1,2,3,4,5]. Among numerous battery systems, lithium-ion batteries (LIBs), due to their high energy density and long cycle life [6,7], have become the dominant technology in the fields of consumer electronics and electric vehicles [8,9,10]. However, LIBs rely on rare metal resources such as lithium and cobalt, and suffer from issues such as high costs, restricted supply chains, and the risk of thermal runaway [11,12]. In addition, emerging systems such as sodium-ion batteries (SIBs) and potassium-ion batteries (PIBs) have certain advantages in terms of resource abundance, but their liquid electrolytes still face safety hazards such as flammability [13] and leakage [14,15,16]. Therefore, the development of energy storage systems that combine safety, sustainability, and economic efficiency has become the core direction of next-generation battery research [17,18].

Against this backdrop, AZIBs have attracted significant attention due to their unique material properties [19,20,21,22]. AZIBs, which use an aqueous solution as the electrolyte, fundamentally eliminate the flammability problem of organic electrolytes, thus significantly enhancing system safety [23]. In comparison with other battery systems, the zinc metal anode of AZIBs has distinct advantages. It has a low standard electrode potential (−0.768 V vs. SHE), superior specific capacity (820 mAh g^−1^), high theoretical volume capacity (5855 mAh cm^−3^), and a large hydrogen evolution overpotential [24,25,26,27,28], good deposition/stripping reversibility, and low cost, considering the abundant zinc reserves on Earth [24]. Moreover, the hydrogen evolution reaction can be suppressed on the anode surface by a passivation layer, which further extends the cycle life. Furthermore, cathode materials such as manganese-based oxides [29,30,31] and Prussian blue analogs [32,33] also possess the characteristics of environmental friendliness and low cost. Therefore, AZIBs are regarded as one of the ideal candidates for constructing large-scale energy storage systems, such as power grid peak-shaving and home energy storage [34,35].

**Figure 1:**
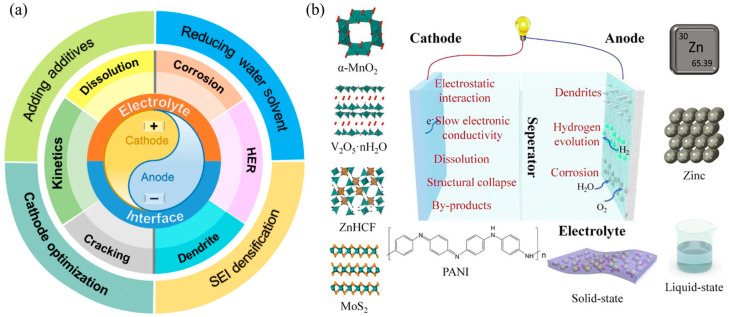
(**a**) Schematic illustration of challenges at the electrode–electrolyte interface in AZIBs and strategies for addressing the challenges [45]. (**b**) Configuration of the AZIBs and corresponding challenges of cathode and anode [28].

Despite the great potential of AZIBs, their commercialization process still faces multiple technical bottlenecks [36,37], as shown in Figure 1a. Firstly, the non-uniform growth of zinc dendrites may lead to short-circuits and even thermal runaway, limiting their cycling stability [38,39,40,41]. Secondly, it is difficult for traditional electrode preparation processes to achieve multi-scale structural design, resulting in low ion-transport efficiency and insufficient utilization of active materials [42,43]. In addition, side reactions at the electrolyte–electrode interface, such as water molecule decomposition and zinc corrosion, will accelerate capacity fading as shown in Figure 1b. These abovementioned problems seriously restrict the application of AZIBs in scenarios requiring high energy density and long lifespan. There is an urgent need to achieve breakthroughs through the synergy of material innovation and structural optimization [44].

In recent years, 3D printing technology (3DP), as a cutting-edge approach in additive manufacturing [46], has provided a novel perspective for the structural design and performance enhancement of AZIBs [47,48]. By depositing materials layer by layer, 3D printing can precisely construct complex three-dimensional structures (such as porous skeletons [49,50], hierarchical pores [47], and heterogeneous interfaces [49]), thereby achieving the following functions: alleviating the growth of zinc dendrites, optimizing the ion-transport pathways [51], enhancing mechanical stability [52], and enabling customized manufacturing. Although some research has made progress in material adaptability, process parameter optimization, and performance testing of 3D-printed AZIBs, its systematic application remains in the exploratory stage. For example, how to reconcile printing precision with large-scale production efficiency, and how to synergistically modulate electrochemical and mechanical properties via structural design [53]. These issues urgently need in-depth analysis from multiple dimensions of materials, structures, and processes.

This paper aims to comprehensively review the latest research progress of 3D printing technology in AZIBs, with a focus on analyzing its mechanism of action in aspects such as electrode structure design, zinc deposition behavior regulation, and interface engineering. Firstly, we will outline the basic principles of 3D printing technology and its applicability in the battery field. Subsequently, from the perspectives of electrode structure innovation, zinc dendrite suppression strategies, and electrolyte–electrode interface optimization, we will systematically summarize the paths for 3D printing technology to improve the performance of AZIBs. Finally, we will discuss the limitations of current research (such as material printability adaptability, process stability, long-term cycling reliability) and future development directions (such as multi-material collaborative printing, intelligent process control). By integrating the synergistic effects of structural innovation and performance optimization, this paper hopes to provide theoretical support and technical guidance for promoting the transition of AZIBs from laboratory research to industrial applications.

## 2. Fundamentals of 3D Printing Technology

Three-dimensional printing technologies, especially material extrusion and vat photopolymerization, have demonstrated great potential in multiple fields, particularly in biomedicine and energy storage. The following will explore the progress and challenges of these technologies in the applications of AZIBs and biomaterials.

### 2.1. Overview of 3D Printing Technology

Three-dimensional printing, also known as additive manufacturing, constructs three-dimensional objects by layer-by-layer material deposition and has become a rapidly evolving manufacturing field [54]. Among them, material extrusion (such as Direct Ink Writing and Fused Filament Fabrication) and vat photopolymerization (such as Stereolithography and Digital Light Processing (DLP)) are two primary 3D printing techniques [55,56].

#### 2.1.1. Direct Ink Writing (DIW)

**Figure 2:**
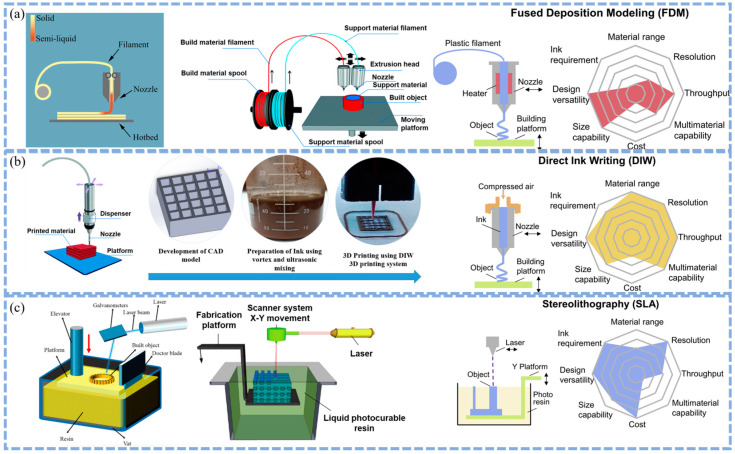
(**a**) Schematic illustration of Fused Filament Fabrication (FFF) technology, showing key components including extrusion head, filament spools (build/support materials), nozzle, hotbed, and moving platform. The process highlights material versatility with plastic filaments and multi-material capability. (**b**) Configuration of DIW system, depicting compressed air-driven dispenser, functional ink preparation (vortex/ultrasonic mixing), nozzle deposition mechanism, and platform movement. The system integrates CAD model development with rheological ink requirements. (**c**) Schematic diagram of SLA apparatus, featuring laser galvanometer system, photocurable resin vat, elevator platform, doctor blade for resin recoating, and X–Y scanning components. The process emphasizes high-resolution capabilities through photopolymerization mechanisms [48,77,96,97].

Direct Ink Writing, an advanced additive manufacturing technology, extrudes viscous inks or slurries through a specially designed nozzle via a compressed-air-driven system and constructs three-dimensional structures in a layer-by-layer manner [56,57,58,59] as shown in Figure 2a. This technology is particularly suitable for the processing and shaping of heat-sensitive materials, enabling the precise fabrication of components with complex geometric configurations. The core principle of DIW technology lies in the utilization of the shear-thinning property of paste-like inks. Under the action of external shear stress, the viscosity of the ink rapidly decreases, allowing for smooth extrusion. Once the shear stress disappears, the ink quickly reverts to a high-viscosity state, ensuring the stability of the formed structure [60,61,62].

From the perspective of material properties, the inks used in the DIW process need to possess appropriate rheological properties, especially a sufficiently high yield stress and storage modulus, to meet the requirements of maintaining the shape of the extruded lines and achieving deformation-free bridging of suspended structures [63,64,65]. With the collaborative effect of Computer-Aided Design (CAD) and control systems, the DIW process can achieve the precise superposition of continuous printed layers, ultimately forming columnar or other complex three-dimensional structures. Thanks to its significant advantages such as a wide range of material selection and high filling rate, DIW technology has demonstrated great application potential in the field of energy storage and has been successfully applied to the fabrication of high-performance batteries [3,66,67] and supercapacitors [68].

Compared with traditional ink-jet printing technology, the DIW process exhibits remarkable technical advantages. Due to the use of paste-like materials with good plasticity, the probability of nozzle clogging during the DIW process is significantly reduced, effectively improving the stability of the printing process and the reliability of the process. This provides a solid guarantee for the efficient and high-quality manufacturing of complex three-dimensional structures [59].

#### 2.1.2. Fused Filament Fabrication (FFF)

Fused Filament Fabrication is a commonly used 3D printing technology [69]. It constructs three-dimensional objects layer by layer by heating and melting thermoplastic materials as shown in Figure 2b, including polymers, metals, ceramics, composites, etc. [70]. In the FFF process, the material is heated and melted in the nozzle [71]. Then, according to the digital model, it is extruded along a specific trajectory to a designated position, where it quickly solidifies and adheres to the surrounding materials. Layers are stacked one by one until the object is completed [72,73,74].

Polylactic Acid (PLA) is one of the most frequently used materials in FFF technology. It has advantages such as non-toxicity, a low melting point (about 176 °C), and good biocompatibility [75,76,77,78,79]. To further improve the performance of FFF-printed parts, research mainly focuses on two directions. One is the optimization of process parameters to enhance the bonding and mechanical properties at the material interface [74,76,77,80]. The other is the modification of PLA. By increasing the degree of crystallization and interactions of PLA molecular chains, its mechanical properties can be improved, and thermal stability, electrical conductivity, and antistatic properties can be imparted to PLA components [75,81,82,83,84].

The FFF technology has also been applied to the manufacturing of battery electrodes [85,86]. For example, 3D printing of LiFePO4 (lithium iron phosphate)/graphite batteries can be achieved through FFF [86]. Mixing lithium iron phosphate (LFP) with PLA to make consumables suitable for FFF printing enables the fabrication of the positive electrode of lithium-ion batteries. This approach provides greater flexibility in battery design, allowing for the direct integration of batteries into 3D-printed objects. The application of 3D printing technology in electrode manufacturing makes it possible to design more complex and efficient battery structures [85].

#### 2.1.3. Stereolithography (SLA)

Stereolithography is a well-established 3D printing technology. It builds three-dimensional objects layer by layer through the photopolymerization of a photosensitive resin [87,88]. In the SLA process, a vat contains a liquid photosensitive resin. A laser beam, controlled by a digital model, traces the cross-sectional pattern of each layer onto the surface of the resin as shown in Figure 2c. When the laser irradiates the resin, the resin undergoes a photochemical reaction and solidifies instantaneously at the irradiated areas. As one layer is completed, the build platform is lowered by a precise distance, and a new layer of resin is spread over the previously solidified layer. The laser then traces the pattern for the next layer, and this process is repeated until the entire 3D object is formed.

One of the key advantages of SLA is its high precision and excellent surface finish [89]. It can produce parts with fine details and smooth surfaces, making it suitable for applications where high-quality esthetics and accurate geometries are required, such as jewelry casting [90,91], dental applications [92], and the production of prototypes for intricate mechanical parts [93]. The photosensitive resins used in SLA offer a wide range of mechanical properties, from flexible to rigid, allowing for customization based on specific application needs. However, SLA also has some drawbacks. The cost of photosensitive resins is generally relatively high compared to some other 3D printing materials [94]. Additionally, post-processing steps, such as cleaning the printed part from excess resin and curing it further under ultraviolet light, are often necessary [95].

### 2.2. Comparison of the Advantages and Disadvantages of Different 3D Printing Technologies (DIW vs. FFF vs. SLA) for Battery Components

The core strengths of DIW lie in its exceptional material compatibility and structural design freedom. It can handle high-viscosity pastes, gels, and suspensions, and is compatible with a wide range of active electrode materials such as LFP, LTO, selenium, rGO, and electrolyte materials like LLZTO ceramic electrolytes and ETPTA/PVDF-HFP polymer electrolytes. This provides a broad space for customized battery material formulations. The most significant highlight of DIW is its ability to precisely manufacture complex three-dimensional geometries, such as high-porosity electrodes, internal interdigitated structures, and vertically aligned ion transport channels. This greatly optimizes ion and electron transport pathways, enhances the loading and utilization of active materials, and promotes electrode–electrolyte interface contact. As a result, it has significant advantages in improving energy density, power density, and rate performance, such as ion transport networks in all solid-state batteries [98]. DIW can achieve high printing precision at the micron level, with the smallest nozzle diameter reaching around 30 μm. It also supports multi-material integrated printing, such as simultaneous printing of anode, cathode, and electrolyte layers, reducing assembly steps and enhancing overall mechanical stability as shown in Table 1.

However, the widespread application of DIW is limited by the stringent rheological requirements for inks. The need for precise thixotropy and viscosity to ensure extrudability and shape retention restricts the range of material choices and increases the complexity and cost of ink formulation development [99]. Printing precision at the micro-nano scale may be limited by nozzle size and ink spreading effects, affecting the realization of ultra-fine structures such as uniform thin films. Structural integrity issues, such as shrinkage and stress caused by drying and curing during printing, as well as potential mechanical strength deficiencies and tolerance to cyclic stress in multi-layered thick structures, especially in flexible batteries, are also key concerns. Additionally, the layer-by-layer deposition and curing requirements result in relatively slow printing speeds, making it challenging to meet large-scale production demands [100].

The primary appeal of FFF lies in its outstanding cost-effectiveness, ease of operation, and widespread accessibility, making it an ideal choice for laboratory prototyping, small-scale production, and educational purposes. It offers a high degree of structural customization, enabling the easy manufacturing of complex-shaped battery casings, supports, and components for thermal management systems [101]. This meets the unique shape requirements for batteries in applications such as wearable devices and the Internet of Things. FFF is also valuable for fabricating molds or templates for the formation of other battery components. By developing composite materials, such as incorporating conductive fillers like CNT and graphene into PLA or ABS, or using high-performance engineering plastics like PEEK or ULTEM, the FFF material library is expanded. This allows for the printing of conductive current collectors or components with specific functionalities, such as thermal conductivity. Adjustments to printing parameters, such as layer thickness, infill density, and orientation, also provide a certain degree of control over mechanical properties.

However, FFF has significant drawbacks in the manufacturing of core battery components. Printed components exhibit marked mechanical anisotropy, with weaker interlayer bonding compared to in-plane strength. This can lead to delamination and failure under cyclic stress during battery operation, threatening long-term stability and safety [102]. Precise control of porosity and density is extremely challenging. Gaps between layers and lines can reduce the effective density and conductivity of electrodes, affecting ion transport and electrolyte wettability. Material selection is severely limited to thermoplastic polymers and their composites [103]. Uniformly dispersing high proportions of functional active materials, such as metal oxides and ceramics, while maintaining their electrochemical activity within a thermoplastic matrix, is a significant technical challenge. High surface roughness can lead to poor internal interface contact, increasing internal resistance. Lower dimensional accuracy makes it difficult to meet the requirements for high energy density and miniaturization [104,105,106]. Thermal effects, such as warping and contraction, can affect the dimensional accuracy and functionality of larger or more complex-shaped components as shown in Table 1.

The greatest strength of SLA is its unparalleled printing precision and excellent surface finish, as well as the ability to manufacture complex internal geometries such as microfluidic channels and fine meshes as shown in Table 1. This makes SLA extremely suitable for producing components that require ultra-high dimensional accuracy and smooth surfaces, such as precision channels in microfluidic batteries, complex microstructures of solid-state electrolytes, or thin-layer interfaces that need precise control. These features help optimize ion transport efficiency and minimize interfacial resistance. SLA can also produce optically transparent components, providing unique convenience for in situ observation of internal battery processes such as lithium dendrite growth. Through resin modification, photo-sensitive materials with certain levels of electrical conductivity, ionic conductivity, or specific mechanical properties can be developed. For example, polyurethane acrylate (PUA)-based gel polymer electrolytes can be used for printing functional battery components, especially solid or gel polymer electrolyte layers. Studies have shown that they can provide uniform ion transport pathways and improve the uniformity of deposition. However, the application of SLA is fundamentally limited by its material system; battery components must be made from photocurable resins, which greatly restricts the direct use of high-proportion, high-performance active electrode materials. These materials usually need to be compounded with photo-sensitive resins, at the expense of some electrochemical performance [106]. The post-processing procedure is complex, requiring cleaning of uncured resin and additional UV or thermal post-curing. This increases time and cost and may introduce defects. The mechanical properties of printed components, such as strength and toughness, as well as their long-term chemical and electrochemical stability, may not match those of components manufactured by traditional methods. They are prone to cracking and failure under volume changes and stress during charging and discharging, which affects cycle life. The limited penetration depth of UV light can lead to uneven internal curing when manufacturing thick-walled components, affecting structural integrity and performance uniformity. Moreover, the cost of equipment and materials is usually higher than that of FFF, reducing the cost-effectiveness for large-scale applications [76].

In summary, these three technologies hold great potential in battery design and manufacturing, such as customized structures, optimized transport, and integrated integration. However, each faces core challenges: DIW is limited by ink formulation and structural stability; FFF is constrained by material limitations and anisotropy; SLA is restricted by photosensitive material selection and component durability. The choice of technology should be based on a comprehensive consideration of specific battery components (such as active electrodes, electrolytes, structural components), performance requirements (such as energy and power density, life, safety), production scale (such as prototyping and mass production), and cost budget. At present, DIW is the most actively studied in the printing of active materials and complex structured electrodes and electrolytes, FFF is more applied in structural components and conductive composite materials, and SLA has unique value in ultra-high precision microstructures and transparent or modified electrolytes.

## 3. Application of 3D Printing Technology in AZIBs

3D printing technology has brought a revolutionary change to the design and manufacture of AZIBs, especially in terms of cathodes, anodes, electrolytes, separators, and the packaging of full-cells. This technology enables customized design, improves material utilization, and simplifies the battery manufacturing process, thus promoting the development of AZIBs.

### 3.1. Cathode Design and Fabrication

#### 3.1.1. Cathode Materials

**Figure 3:**
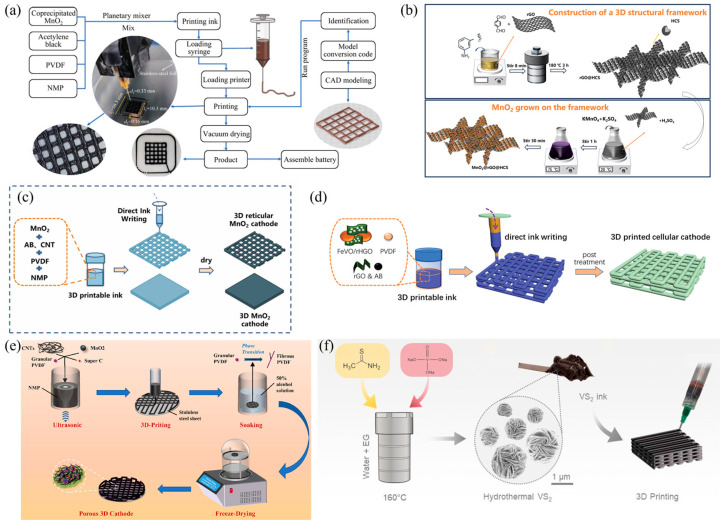
(**a**) Schematic diagram of DIW process and photograph of printed product [107]. (**b**) Schematic illustration of the synthesis process of rGO@HCS and MnO2@rGO@HCS [108]. (**c**) DIW process diagram of 3D reticular MnO_2_ cathode and 3D MnO_2_ cathode [109]. (**d**) Schematic of DIW-based fabrication of cellular FeVO/rHGO cathodes for ZIBs [35]. (**e**) Schematic illustration of the fabrication process of high MnO_2_ mass loading 3D cathode [110]. (**f**) Schematic showing the synthesis of the hydrothermal VS_2_ microflowers, the formulation of VS_2_ inks and the DIW of VS_2_ inks to fabricate battery and supercapacitor electrodes [111].

3D printing technology can be used to prepare cathode materials with specific structures, such as MnO_2_, through methods like DIW. Research by Liu et al. indicates that customized 3D-printed MnO_2_ cathodes can overcome the issues of poor cycling stability and slow ion diffusion in traditional MnO_2_ cathodes as shown in Figure 3a. By precisely controlling the rheological properties of the ink, the reticulated layer structure after printing can be maintained, thus improving the battery’s cycling performance [107].

In addition, the Ding research group discovered that 3D printing can also be employed to construct composite cathode materials as shown in Figure 3b They proposed a MnO2@rGO@HCS cathode material, which features a uniquely ordered 3D hierarchical framework synthesized by the hydrothermal method. The non-template in situ growth of hollow carbon spheroids (HCS) on reduced graphene oxide (rGO) creates a comprehensive ordered channel network that can serve as a “highway” for electrolyte transport. Then, MnO_2_ nanoparticles are uniformly deposited within this framework, forming numerous “service stations” that provide ample ion storage sites along the transport path. This architecture not only accelerates ion transport but also significantly enhances ion storage capacity [108]. Peng’s designed and fabricated a novel 3D-printed mesh-like MnO_2_ self-supported cathode (DIW). The AZIB (Alkali-Zinc-Ion Battery) achieved a high areal capacity of 3.43 mAh cm^−2^ at 2 mA cm^−2^, thanks to the high mass loading and mesh-like structure of the MnO_2_ cathode. The mesh-like structure facilitated electrolyte storage and rapid diffusion of Zn^2+^, and reduced the charge transfer impedance. Moreover, the 3D printing technique enabled layer-by-layer printing of electrodes. The areal capacity of the double-layer 3D mesh MnO_2_ cathode was nearly twice that of the single-layer 3D mesh MnO_2_ (Figure 3c). This work demonstrates the great potential of 3D printing technology in designing and manufacturing high-loading electrodes with high capacity and customized shapes [109].

Ma et al. utilized DIW to print a hierarchically porous FeVO/rHGO cathode with a porosity of 72% (Figure 3d). The honey-comb-like structure enhances the electrode integrity through the mechanical interlocking effect. When the mass loading is 24.4 mg/cm^2^, the areal capacity reaches 7.04 mAh/cm^2^, and the capacity retention rate is 91% after 650 cycles [35]. The unique structure of the 3D-printed composite cathode provides interpenetrating pathways for the transport of both electrons and ions. The outstanding electrochemical performance paves new ways for designing state-of-the-art cathodes for ZIBs.

Nie et al. used ultrasonication to blend materials including carbon nanotubes (CNT), carbon black (Super C), MnO_2_, and poly(vinylidene fluoride) (PVDF) to form a printable ink, which was then printed as a pre-designed multilayer mesh structure on a stainless steel plate (DIW). After subsequent freeze-drying treatment, a mesh-structured cathode with a high MnO_2_ mass loading for AZIB was obtained, denoted as the 3D cathode (Figure 3e). The mass loading of this cathode can be conveniently adjusted by changing the number of printed layers. Moreover, the mesh structure endows the cathode with a considerable surface area, promoting enhanced active site availability for reactions during charging/discharging and enhancing the pseudocapacitive phenomenon. Notably, the stable and uniform microstructure not only accelerates ion/electron transport but also promotes the stability of the AZIB. Therefore, the 3D cathode exhibits outstanding comprehensive electrochemical performance [110].

Tagliaferri et al. created a hierarchical pore network (with pore diameters of 1–5 μm) by printing a porous micrometer-pillar structure based on DIW technology and combining it with a VS_2_ micro-flower ink synthesized through a thermal process (Figure 3f). This structure shortens the diffusion path of Zn^2+^ (increasing the diffusion coefficient to 2.1 × 10^−9^ cm^2^/s) and enhances the surface adsorption capacity. As a result, it achieves an areal capacity of 1.98 mAh/cm^2^ at a high voltage of 1.5 V and retains 65% of its capacity after 100 cycles [111].

#### 3.1.2. Anode Materials

In recent years, with the increasing demand for high-energy-density and low-cost battery systems, AZIBs have received significant attention due to their safety, environmental friendliness, and abundant resources. However, the practical applications of AZIBs are still limited by the performance of zinc anodes, especially the reversibility and cycling stability under conditions of high zinc utilization. Traditional two-dimensional zinc foil anodes often exhibit poor electrochemical performance and a limited lifespan due to issues such as zinc dendrite growth, volume expansion, and side reactions [112,113].

**Figure 4:**
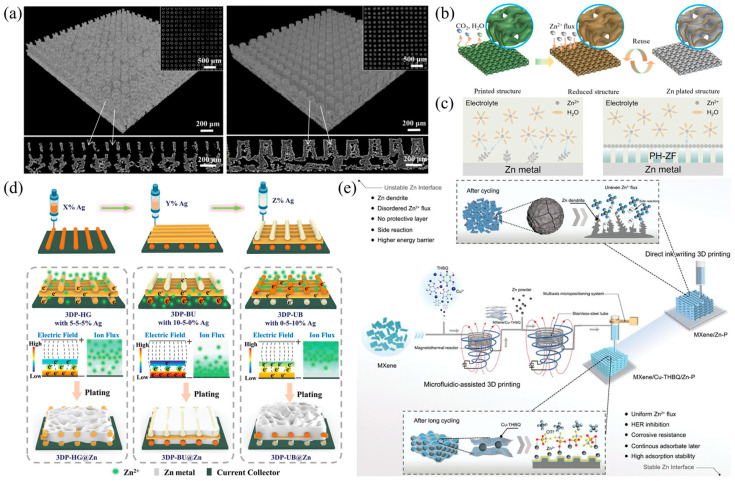
(**a**) Schematic demonstration of the tubular and pilar arrays [114]. (**b**) Schematic demonstration of the procedures for preparing reusable 3DP-rGG@Zn anode [47]. (**c**) Schematic illustration of Zn plating on bare Zn and PH-ZF coated Zn anode [49]. (**d**) Schematic demonstration of the process of fabricating 3D-printed structures with varying silver (Ag) content and their subsequent electrochemical behavior [115]. (**e**) Schematic of the difference between M3DP technology and traditional direct-ink-writing printing and the morphology evolution of the printed zinc anode during Zn stripping/plating cycling [116].

To address the above-mentioned issues, Wu et al. proposed an innovative strategy as shown in Figure 4a. They prepared graphene arrays (including tubular and pilar arrays) through 3D printing technology (DLP) which solved the problems of dendrite growth, volume expansion, and side reactions of traditional two-dimensional zinc foil anodes under high zinc utilization. Experimental results show that the symmetric battery with a 3DGs@Zn anode has a lifespan of up to 1100 h at a current density of 2 mA cm^−2^. The pouch-type battery achieved a zinc utilization rate of 47.12% and an areal capacity of 3.76 mAh cm^−2^. These results significantly improve the performance and practicality of AZIBs, providing important reference for the next-generation battery technology [114].

Yu et al. utilized 3D printing technology to fabricate a 3D gyroid structure based on reduced graphene oxide as the zinc anode framework, which enhanced the utilization rate of zinc as shown in Figure 4b. By means of digital light processing (DLP) 3D printing technology, a three-dimensional helical structure based on reduced graphene oxide (3DP-rGG) was prepared as the zinc anode framework. This structure can effectively regulate the distribution of local current density, provide sufficient nucleation sites and free space, thereby enabling uniform zinc deposition and accommodating tiny zinc nodules. The results indicate that the 3D-printed graphite framework exhibits remarkable reversibility during the zinc electroplating and stripping processes, featuring high coulombic efficiency and low voltage hysteresis. The full- cell tests demonstrate its high specific capacity and excellent long-term cycling stability. Additionally, the 3DP–rGG structure can be reused more than ten times without affecting its electrochemical performance, demonstrating its potential as an efficient and controllable method for preparing 3D graphite current collectors [47].

In addition, a polymer coating can be prepared on zinc metal via 3D printing technology (DIW) to promote the uniform deposition of zinc ions, thereby improving the cycling performance of the battery as shown in Figure 4c. The Zhang research team proposed a new strategy of constructing a ferroelectric porous PVDF-HFP protective layer on the surface of a zinc anode through 3D printing technology. This effectively addresses the serious problems of zinc dendrite growth and side reactions in AZIBs. Through the synergistic effect of zinc salts and 3D printing, not only the transformation of the polymer phase structure and the formation of a porous surface are achieved, but also the uniform deposition of Zn^2+^ and ion migration are promoted, significantly enhancing the cycling stability and rate performance of the battery. Experimental results show that the symmetric battery exhibits long-cycle lifetimes of 1200 h and 2000 h at current densities of 0.5 mA cm^−2^ and 1.0 mA cm^−2^, respectively. The full-cell maintains a high specific discharge capacity of approximately 88.3 mA g^−1^ after 1000 cycles at 1.0 A g^−1^. This method provides a simple and efficient approach to improving the performance of zinc-ion batteries [49].

Three-dimensional printing can also be utilized to construct a three-dimensional porous zinc metal anode. By designing an electron/ion flux gradient, the growth of zinc dendrites can be inhibited. He et al. designed a 3D porous zinc anode with a dual-gradient electron/ion flux (3DP-BU@Zn) through 3D printing technology (DIW), effectively solving the problem of dendrite growth in zinc-ion batteries, which is caused by the concentration of ions at the top and the random distribution of electrons as shown in Figure 4d. This anode establishes a bottom-up gradient electron flux by being rich in conductive silver nanoparticles at the bottom, and realizes a downward ion pumping effect using zincophilic silver nanoparticles. Meanwhile, the hierarchical porous structure and continuous conductive network endowed by 3D printing ensure efficient electron transfer and ion diffusion, thus achieving a bottom-priority zinc deposition behavior. Experimental results demonstrate that the 3DP-BU@Zn symmetric battery exhibits a minimal voltage hysteresis of 17.7 mV and an excellent cycling life of over 630 h under the conditions of 1 mA cm^−2^ and 1 mAh cm^−2^. The full-cell also shows remarkable stability during 500 cycles, providing important reference for the next-generation safe and durable zinc-metal batteries [115].

Through microfluidics-assisted 3D printing technology (DIW), He et al. fabricated a zinc-powder anode, whose surface was covered with a two-dimensional heterostructured functional layer composed of MXene and Cu-THBQ as shown in Figure 4e. Relying on its excellent anti-corrosion performance and strong adsorption capacity for zinc ions, this functional layer effectively regulated the zinc-ion deposition behavior and inhibited the hydrogen evolution reaction (HER), thus significantly enhancing the cycling stability of the zinc anode. Experimental results show that the symmetric battery based on this anode can operate stably for 1800 h under the conditions of 2 mA cm^−2^/1 mAh cm^−2^. Moreover, the full-cell paired with an organic cathode exhibits high reversible capacity and long-cycle life, providing technical support for the design of high-performance zinc–organic batteries [116].

### 3.2. Electrolyte and Separator

Electrolytes and separators are crucial components in AZIBs, influencing the ionic conductivity, safety, and cycle life of the batteries. However, traditional separators have not been adequately studied. Three-dimensional printing technology offers the possibility of fabricating electrolytes and separators with specific structures and functions [117].

**Figure 5:**
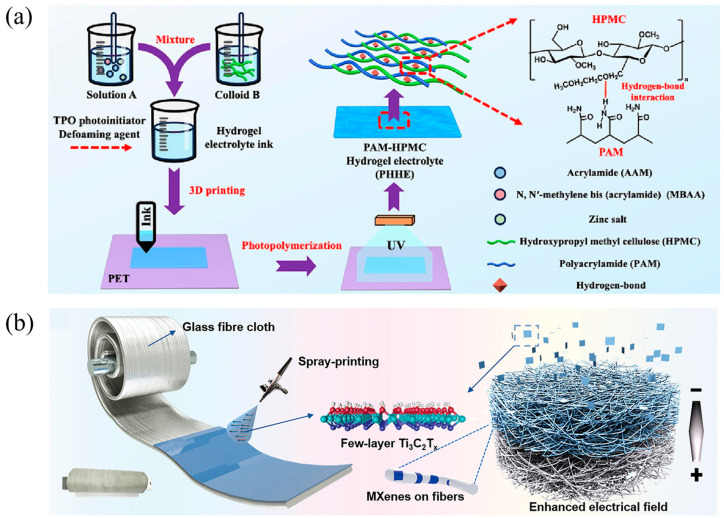
(**a**) Preparation process of the PHHE [118]. (**b**) Schematic diagram depicting the preparation and function of MXene-GF separator [120].

Lu et al. developed an electrolyte material based on a double-network crosslinked hydrogel, which features good ionic conductivity (31.72 mS cm^−1^), a wide voltage window (0–2.3 V), and excellent mechanical flexibility as shown in Figure 5a. It is suitable for the integrated design of electrolyte and separator in flexible zinc-ion microbatteries (FZIMBs). By utilizing DIW, this method can fabricate complex structures that are difficult to achieve with traditional templating methods, overcoming the limitations of conventional approaches in manufacturing two-dimensional and simple three-dimensional structures. This not only enhances the energy density and cycling stability of the battery but also demonstrates great application potential in wearable electronic devices [118].

Poompiew et al. successfully fabricated polyacrylamide (PAM)-based hydrogel polymer electrolytes (HPEs) with controllable porosity using DLP 3D printing technology. The sample with 40% porosity exhibited optimal electrochemical performance, including an ionic conductivity of 28.10 mS cm^−1^, a specific capacity of 161.4 mAh g^−1^, and good cycling stability. By employing DLP technology, they were able to precisely control the structure and porosity of the PAM-based hydrogel, which is crucial for optimizing the ionic transport properties and mechanical stability of the electrolyte. Compared to traditional electrolyte preparation methods, this approach may offer significant advantages in customization and production efficiency. By altering the porosity of the hydrogel, researchers can explore its effects on ionic conductivity, electrochemical stability, and mechanical strength, thereby providing design insights for the development of high-performance flexible batteries [119].

Su et al. introduced a scalable Janus separator modified with Ti_3_C_2_Tx MXene, which was prepared by ink-jet printing MXene nanosheets onto commercial glass fibers. This separator features a high dielectric constant (an optimized value of 53.5), which can provide a directional electric field to accelerate the zinc-ion flux and repel anions (Figure 5b). Consequently, it effectively inhibits the growth of zinc dendrites and side reactions, enabling a stable zinc anode. The symmetric battery using this separator can achieve long-term stable cycling under different current densities (e.g., 1180 h at 1 mA cm^−2^ and 1200 h at 5 mA cm^−2^). Moreover, the aqueous zinc-ion full-cell with this separator retains 77.9% of its capacity after 1000 cycles at 5.0 A g^−1^ [120].

### 3.3. Full-Cell Packaging

Full-cell packaging is a crucial step in achieving high-performance AZIBs. Three-dimensional printing technology can be employed to fabricate battery casings with specific shapes and structures, enabling the customization and integration of batteries. For instance, through DIW and FFF methods, zinc-ion hybrid capacitors with hierarchical porous micro-lattice cathodes, metal anode stabilizers, quasi-solid-state gel electrolytes, and plastic encapsulations can be 3D-printed, thereby achieving ultra-high areal capacitance and excellent cycling stability [121]. Chen et al. designed and fabricated a flexible quasi-solid-state zinc-ion battery. By using the direct-writing 3D printing technique, highly conductive silver conductive paste was printed onto a PET substrate as a current collector, demonstrating the application of 3D printing technology in the manufacture of flexible energy-storage devices [122].

Zhu et al. utilized the two-electrode printed micro-battery technology. The V_2_O_5_@CNT cathode and zinc-powder anode was simultaneously printed by DIW. Through structural matching (with a spacing of 50 μm), dendrite penetration was inhibited. Encapsulation with a gel electrolyte enabled the micro-battery to achieve an areal capacity of 0.51 mAh/cm^2^ and an energy density of 0.37 mWh/cm^2^ [123].

**Figure 6:**
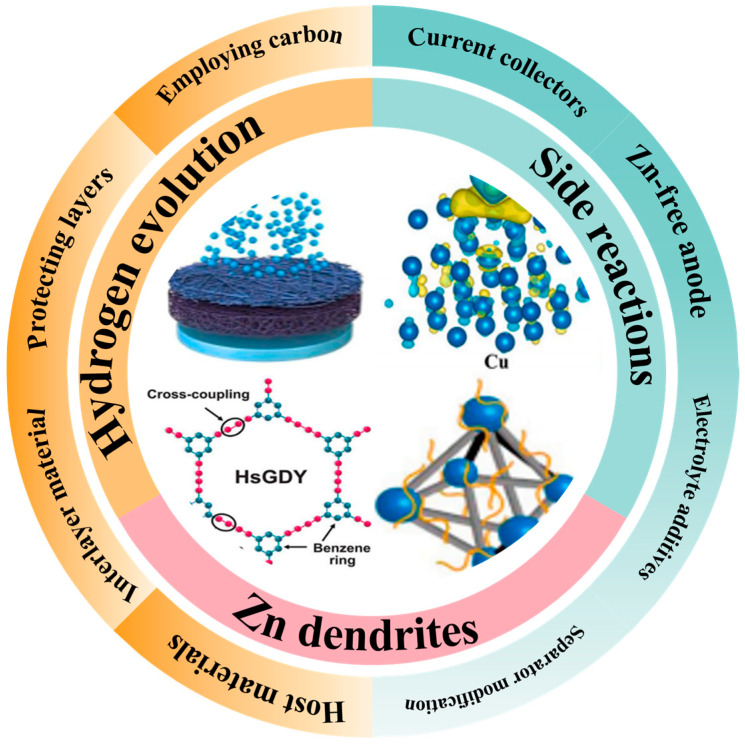
Diagram of the powerful role of carbon materials in AZIBs.

Overall, 3D printing technology offers great flexibility and innovation in the design and manufacture of various components of AZIBs. Through 3D printing, the structures and compositions of cathodes, anodes, electrolytes, and separators can be customized, thus improving the performance and reliability of batteries. In the future, with the continuous development of 3D printing technology, more breakthroughs are expected in the field of AZIBs [3]. As shown in Figure 6, carbon materials and battery enhancement strategies can be utilized to improve the performance of AZIBs [112].

## 4. Challenges of 3D Printing Technology in AZIBs

The application of 3D printing technology in AZIBs has brought new possibilities for battery design and manufacturing, yet it also confronts numerous challenges. The following will provide a detailed analysis of these challenges from aspects such as materials, design, and applications.

### 4.1. Challenges in Terms of Materials

#### 4.1.1. Development of Inks/Slurries

For 3D printing, printable inks or slurries are required. These materials must meet specific rheological properties, such as proper viscosity and thixotropy, to ensure the smooth operation of the printing process and the precision of the printed structure. Nevertheless, the options for electrode, electrolyte, and separator materials suitable for AZIBs are restricted. Developing composite materials that not only exhibit good electrochemical performance but are also compatible with 3D printing represents one of the key challenges [35]. For example, there is a need to design 3D-printable electrolytes featuring high ionic conductivity and robust mechanical strength [124].

#### 4.1.2. Inter-Material Compatibility

In AZIBs, different materials are commonly employed for the cathode, anode, electrolyte, and separator. The chemical and electrochemical compatibility among these materials is of utmost importance. During the 3D-printing process, it is essential to ensure that these materials do not undergo adverse reactions during printing and subsequent use, which could otherwise affect the battery’s performance and lifespan.

#### 4.1.3. Material Adaptability and Modification

Key materials for AZIBs, such as cathode materials like manganese dioxide [109,110] and Prussian blue analogs [125,126], and anode material zinc metal [47,49,127], need to possess suitable 3D-printing properties. This typically requires modifying the materials, for example, by adding conductive agents or binders, to enhance their printability and electrochemical performance [128]. Challenges in terms of design.

### 4.2. Challenges in Terms of Design

#### 4.2.1. Electrode Structure Design

3D printing makes it possible to design electrodes with complex geometries, such as porous structures [35,129] and interpenetrating network structures [130]. These structures can increase the surface area of the electrodes and facilitate ion transport, thereby enhancing the battery’s capacity and rate performance. However, it remains a challenge to design the optimal electrode structure to maximize battery performance.

#### 4.2.2. Battery Integration Design

Three-dimensional printing enables the integration of various battery components into a single, unified structure. This simplifies the battery manufacturing process and enhances both the energy density and power density of the battery. Nevertheless, devising a compact and efficient battery integration solution requires a comprehensive consideration of factors such as the battery’s electrochemical performance, thermal management, and mechanical strength [97].

#### 4.2.3. Customized Design

The most significant advantage of 3D printing lies in its customization capabilities. Through 3D printing, batteries with specific shapes, sizes, and properties can be designed according to different application requirements. However, it remains a challenge to integrate the customization advantages of 3D printing with the practical applications of AZIBs and develop customized battery products that are competitive in the market.

### 4.3. Challenges in Terms of Applications

For the large-scale production of AZIBs, the issue of material costs needs to be addressed first. Although zinc is abundant in resources and relatively low-cost, the costs of cathode materials, electrolytes, and separators still need to be further reduced. In addition, when applying 3D printing technology to battery manufacturing, the suitability of printing materials and printing efficiency need to be considered to meet the demands of large-scale production. Traditional battery production processes are complex and relatively inefficient. Three-dimensional printing technology holds the promise of simplifying the production process and enabling automated manufacturing, yet it currently still faces challenges such as equipment precision, printing speed, and stability. Optimizing 3D printing process parameters to improve production efficiency is the key to achieving large-scale production. Large-scale production requires ensuring the consistency of product quality. For 3D-printed AZIBs, a complete quality control system needs to be established, including raw material inspection, printing process monitoring, and finished-product performance testing, to ensure the stability and reliability of battery performance [97].

## 5. Discussion

The integration of additive manufacturing (AM) into AZIBs represents a paradigm shift in battery design, enabling unprecedented control over electrode architectures, electrolyte–separator interfaces, and full-cell configurations. Compared to conventional fabrication methods, 3D printing offers unique advantages in tailoring hierarchical structures (e.g., porous frameworks, gradient interfaces) that address critical challenges such as zinc dendrite growth, ion transport inefficiency, and interfacial side reactions. For instance, DIW-printed MnO_2_ cathodes with reticulated layers and honeycomb-like FeVO/rHGO composites demonstrate enhanced ion diffusion kinetics and mechanical stability, achieving high areal capacities (>7 mAh/cm^2^) and prolonged cycling stability (>650 cycles). Similarly, 3D-printed graphene or MXene-modified zinc anodes regulate Zn^2+^ deposition behavior, extending symmetric cell lifespans to >1800 h by homogenizing local current density and suppressing hydrogen evolution. These advancements highlight how structural innovations—enabled by AM—synergize with material properties to overcome intrinsic limitations of AZIBs.

However, the translation of lab-scale 3D-printed prototypes to industrial applications faces multifaceted challenges. First, material compatibility remains a bottleneck: printable inks must balance rheological properties (e.g., shear-thinning behavior for DIW) with electrochemical performance. While conductive additives (e.g., Ag nanoparticles, rGO) enhance printability, they may introduce parasitic reactions or compromise energy density. Second, process scalability conflicts with precision. High-resolution techniques like SLA achieve sub-μm features but struggle with throughput, whereas FFF/DIW sacrifices resolution for speed. This trade-off limits the viability of AM for mass production unless hybrid strategies (e.g., multi-nozzle systems, AI-driven parameter optimization) are adopted. Third, long-term reliability under realistic conditions—such as mechanical stress during cycling or electrolyte leakage in flexible configurations—requires further validation. For example, hydrogel electrolytes with double-network structures show promise in suppressing dendrites, but their mechanical degradation after repeated bending remains unaddressed.

Future research should focus on three interconnected axes: multi-material integration, process intelligence, and system-level optimization. Multi-material 3D printing (e.g., co-printing electrodes and solid-state electrolytes) could enable monolithic battery architectures with seamless interfaces, minimizing impedance losses. Machine learning algorithms may accelerate ink formulation and printing parameter optimization by predicting rheological-electrochemical correlations. Additionally, combining AM within situ characterization (e.g., X-ray tomography) will deepen understanding of dynamic structural evolution during cycling, guiding the design of failure-resistant architectures. Ultimately, the commercialization of 3D-printed AZIBs hinges on cross-disciplinary collaboration to align material innovation, structural design, and scalable manufacturing.

## 6. Conclusions

In conclusion, this review presents an overview of the progress in applying 3D printing technology to AZIBs, outlining its potential role in enhancing battery performance via structural innovation and performance optimization. The detailed examination of 3D printing techniques, such as DIW, FFF, and SLA, has revealed their unique advantages and challenges in the context of AZIBs. Through numerous case studies, it is evident that 3D printing can effectively address critical issues such as zinc dendrite formation, ion transport inefficiency, and interface instability, thereby improving the capacity, cycling stability, and safety of AZIBs. However, the successful transition of 3D-printed AZIBs from laboratory research to industrial applications requires overcoming several hurdles, including the development of suitable printing materials, optimization of printing processes, and establishment of quality control systems. Future research should focus on multi-material collaborative printing, intelligent process control, and the exploration of new materials to further enhance the performance and reliability of AZIBs. This review underscores the importance of integrating structural innovation with performance optimization in the advancement of AZIB technology. By addressing the current limitations and leveraging the strengths of 3D printing, it is anticipated that AZIBs will play a pivotal role in meeting the growing demand for safe, sustainable, and cost-effective energy storage systems. The insights and recommendations provided in this review aim to guide researchers and engineers in their efforts to push the boundaries of AZIB technology, fostering breakthroughs that will support the global transition to renewable energy systems.

## Figures and Tables

**Table 1:** Comparison of the Core Characteristics of Mainstream 3D Printing Processes in Battery Manufacturing.

Technology	Core Applicable Battery Components	Electrode Thickness Range	Surface/Layer Accuracy (XY)	Line/Feature Resolution (Min. Line Width)	Core Advantages	Core Limitations
DIW	Thick Electrodes, Porous Structure Electrodes, Solid Electrolyte Structures, Integrated Battery Architectures	200–800 μm	±20–100 μm	100–500 μm	High material freedom, Precise ionic channel construction, Mold-free integrated forming	Electrode cracking due to ink drying shrinkage, Insufficient nanoscale resolution, Slow printing constrains mass production
FFF	Battery Casings/Brackets, Current Collector Molds, Molds for Electrode Casting	50–500 μm (post-casting)	±100–200 μm	200–500 μm	Ultra-low cost, Customized shapes, Conductive composites applicable	Interlayer voids reduce electrode density, Thermoplastic matrix limits active material loading, Anisotropy causes delamination failure
SLA	Microfluidic Electrolyte Layers, Precision Solid Electrolyte Structures, Miniature Battery Components	10–200 μm (single layer)	±5–25 μm	10–100 μm	Submicron accuracy, Smooth interfaces, Potential for in situ monitoring	Photocurable resin restricts material selection, Post-processing (cleaning, curing) risks damaging thin electrolyte layers, Uneven curing within thick electrodes
